# The Protective Effect of Bergamot Polyphenolic Fraction (BPF) on Chemotherapy-Induced Neuropathic Pain

**DOI:** 10.3390/ph14100975

**Published:** 2021-09-25

**Authors:** Sara Ilari, Filomena Lauro, Luigino Antonio Giancotti, Valentina Malafoglia, Concetta Dagostino, Micaela Gliozzi, Antonia Condemi, Jessica Maiuolo, Francesca Oppedisano, Ernesto Palma, Carlo Tomino, Daniela Salvemini, Vincenzo Mollace, Carolina Muscoli

**Affiliations:** 1 Department of Health Science, Institute of Research for Food Safety & Health (IRC-FSH), University “Magna Graecia” of Catanzaro, 88201 Catanzaro, Italy; sara.ilari@hotmail.it (S.I.); c_dagostino@libero.it (C.D.); micaela.gliozzi@gmail.com (M.G.); antonella.condemi@libero.it (A.C.); jessicamaiuolo@virgilio.it (J.M.); oppedisanof@libero.it (F.O.); palma@unicz.it (E.P.); mollace@libero.it (V.M.); 2 Department of Pharmacology and Physiology, Henry and Amelia Nasrallah Center for Neuroscience, Saint Louis University School of Medicine, 1402 S. Grand Blvd, St. Louis, MO 63104, USA; milena.lauro@health.slu.edu (F.L.); luigi.giancotti@health.slu.edu (L.A.G.); daniela.salvemini@health.slu.edu (D.S.); 3 Institute for Research on Pain, ISAL Foundation, Torre Pedrera, 47922 Rimini, Italy; valentinamalafoglia@yahoo.it; 4 Scientific Direction, IRCCS San Raffaele Roma, 00166 Rome, Italy; carlo.tomino@sanraffaele.it

**Keywords:** oxidative stress, chemotherapy-induced peripheral neuropathy (CIPN), natural antioxidants, bergamot polyphenol fraction (BPF), chronic pain

## Abstract

Paclitaxel is a chemotherapeutic drug used for cancer treatment. Chemotherapy-induced peripheral neuropathy (CIPN) is a common major dose-limiting side effect of many chemotherapeutic agents, including paclitaxel. CIPN is accompanied by mechanical and thermal hypersensitivity that resolves within weeks, months, or years after drug termination. To date, there is no available preventive strategy or effective treatment for CIPN due to the fact that its etiology has not been fully explained. It is clear that free radicals are implicated in many neurodegenerative diseases and recent studies have shown the important role of oxidative stress in development of CIPN. Here, we observed how, in rats, the administration of a natural antioxidant such as the bergamot polyphenolic extract (BPF), can play a crucial role in reducing CIPN. Paclitaxel administration induced mechanical allodynia and thermal hyperalgesia, which began to manifest on day seven, and reached its lowest levels on day fifteen. Paclitaxel-induced neuropathic pain was associated with nitration of proteins in the spinal cord including MnSOD, glutamine synthetase, and glutamate transporter GLT-1. This study showed that the use of BPF, probably by inhibiting the nitration of crucial proteins involved in oxidative stress, improved paclitaxel-induced pain behaviors relieving mechanical allodynia, thermal hyperalgesia, thus preventing the development of chemotherapy-induced neuropathic pain.

## 1. Introduction

Chemotherapy-induced peripheral neuropathy (CIPN) is a prominent side effect of many chemotherapy agents. Although researchers have studied CIPN for years, its pathogenesis is not fully understood [1,2]. Chemotherapy agents, designed to eliminate dividing cancer cells, can also damage healthy cells inducing numerous side effects such as drowsiness, fatigue, insomnia, dizziness, nausea, and the onset of CIPN [2,3]. Taxanes (e.g., paclitaxel and docetaxel), platinum-based drugs (e.g., carboplatin, cisplatin and oxaliplatin), vinca alkaloids (e.g., vincristine and vinblastine), epothilones (e.g., ixabepilone), thalidomide, and bortezomib are, probably, the most used chemotherapeutic agents [3]. Taxanes act against solid tumors such as breast, cervical, ovarian cancer, lung cancers, and for Kaposi’s sarcoma [4]. They stabilize the microtubules, thus blocking the cell cycle in the late G2 phase of mitosis, and thus inducing cell death [5,6]. Paclitaxel is one of the most commonly used taxanes in the clinic. It is a natural product isolated from the bark of the Pacific Taxus brevifolia. Although taxanes prolong the survival of cancer patients, they are still associated with a significant onset of toxicity that reduces the quality of life [7]. One of the main taxanes side effects is neuropathic pain [8,9], and the mechanisms responsible for this alteration in sensitivity pain are still unknown [10]. The initiation and progression of CIPN are related to intraepidermal nerve fibers impairment, neuroimmune system activation, proinflammatory cytokine production, and oxidative stress [3,11]. However, oxidative stress, and thus the development of free radicals (ROS/RNS), play a crucial role in the pathogenesis of both degenerative and neurodegenerative diseases [12,13]. ROS is involved in both neuropathic and inflammatory pain conditions [14,15,16,17,18,19,20,21]. A ROS excess builds up in glial cells, leading to neuronal damage and dysfunction.

Additionally, ROS activate second messenger systems involved in the sensitization of dorsal horn neurons and possibly also activate spinal glial cells, which in turn play an important role in chronic pain [22,23,24].

It has been observed that ROS/RNS over-production is responsible for cellular macromolecules’ damage-causing alteration and post-translational modification of key proteins in different animal models of pain, including those of inflammatory pain, oxaliplatin [25] or spinal cord injury (SCI) [26] induced neuropathic pain, diabetic neuropathy [27] or pain induced by chronic administration of morphine [28].

In particular, ROS/RNS over-production induces mitochondrial manganese superoxide dismutase (MnSOD), glutamate transporter (GLT-1), glutamate receptor N-methyl-D-aspartate (NMDA), and glutamine synthase (GS) post-translational modification leading in turn to the alteration of the physiological glutamate turnover with consequent excitotoxicity and enhanced pain response.

ROS/RNS levels are finely controlled by endogenous antioxidant systems [29,30,31]. However, in pathological conditions, free radical levels increase due to their enhanced production or decreased level of endogenous antioxidants [32,33,34,35]. Numerous compounds have been developed to prevent CIPN by blocking ion channels, acting on the development of inflammatory cytokines, and inhibiting oxidative stress [36].

Current therapies, including glutathione or duloxetine, appear to be effective for the prevention and treatment, respectively, of CIPN, but further clinical studies are necessary regarding the type of chemotherapy agents used and their side effects [3,37]. In addition, preclinical studies investigated the natural or synthetic antioxidant effect against the pain of different etiologies and the prevention of CIPN [15,28,38,39,40]. In fact, we identified that overexpression of peroxynitrite, the activation of nitric oxide synthase and NADPH oxidase, and the inhibition of manganese superoxide dismutase (MnSOD), after intraperitoneal administration of paclitaxel in rats, lead to neuropathic pain together with an increase of TNF-α, IL-1β, and nitration of GLT-1 and GS [25]. Removing PN with peroxynitrite decomposition catalysts, such as FeTMPyP5+ and MnTE-2-PyP5+ [25], blocked neuropathic pain without interfering with antitumor effects.

Medicinal plants thanks to the presence of different phytochemicals, including polyphenoid and flavonoids, have anti-inflammatory and antioxidant effects [41,42] and therefore have shown beneficial properties on various neurological and psychiatric diseases such as mild cognitive impairment, epilepsy, Parkinson’s disease [43], cancer and cardiovascular disease [41]. In particular, flavonoids are a family of polyphenols found mainly in fruit and vegetables. Their bioactivity has been related to their antioxidant effects, thus preventing oxidative stress (which is thought to be one of the causes of disorders affecting the central nervous system) [44]. It has been reported that flavonoids have the ability to cross the blood-brain barrier [45,46,47,48]. Their beneficial activities are, most probably, due to their antioxidant properties together with their modulation of the intracellular pathways responsible for the activity within the brain, including the regulation of cell survival and mitochondrial function [45,49].

Bergamot is an endemic plant with a high content of flavonoid and glycosides, including naringin, neoeriocitrin, and rutin. Poliphenolic fraction of bergamot (BPF) demonstrated a high capacity to inhibit LDL oxidation [50,51]. Bergamot also possess a protective role in patients with metabolic syndrome and cardiometabolic risk [52]. Furthermore, administration of bergamot juice in rats on a hypercholesterolemic diet led to a reduction in fatty liver disease, although the mechanism is not yet clear [52].

New scientific evidence also supports the efficacy of natural agents in the management of the pain of different etiologies. Polyphenols, such as bergamot, oleuropein, and resveratrol, have strong antioxidant and analgesic properties [38,39,53,54,55,56].

Our recent studies showed the antioxidant protective effect of bergamot polyphenolic fraction (BPF) during neuropathic pain induced by sciatic nerve ligation in rats [39]. The administration of BPF attenuated the allodynia and hyperalgesia measured during the development and maintenance of neuropathic pain by ROS/RNS removal [39].

Therefore, medicinal plants constitute valuable new options for CIPN management [54,57]. Based on these data, we propose the use of BPF as add-on therapy to inhibit the paclitaxel-induced pain behaviors by relieving mechanical allodynia, thermal hyperalgesia, and nitration of crucial proteins involved in oxidative stress, thus preventing the development of chemotherapy-induced neuropathic pain.

## 2. Results

### 2.1. BPF Effects on Paclitaxel-Induced Thermal Hyperalgesia and Allodynia

**Figure 1:**
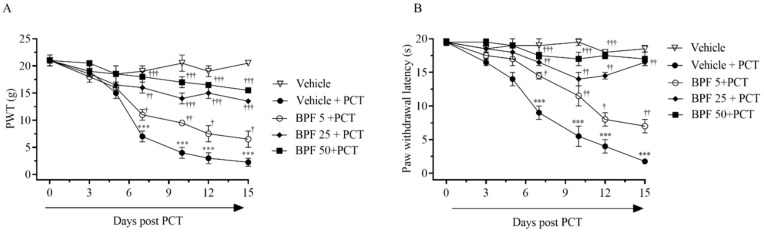
Paclitaxel-induced mechano-allodynia (**A**) and thermal hyperalgesia (**B**) was inhibited by intraperitoneal (i.p.) administration of BPF (5–50 mg/Kg/day; 15 min prior to each paclitaxel injection). Paclitaxel (2 mg/Kg/die) and BPF were injected in rats on alternate days, until D15 (D0,3,5,7,10,12,15). Behavior measurements were assessed on D0, prior to injection, D3, D5, D7, D10, D12, and D15. Results are expressed as a mean ± SD. †: *p* < 0.05; ††: *p* < 0.01; †††: *p* < 0.001 compared vs. paclitaxel (PCT), and ***: *p* < 0.001 compared vs. vehicle.

Based on our previous data [25], using a known paclitaxel-induced neuropathic pain model, we confirmed that intraperitoneal (i.p.) administration of paclitaxel (PCT) in rats, for 15 alternative days (D), produced a development of mechanical allodynia (Figure 1A) and thermal hyperalgesia (Figure 1B) (neuropathic pain). As expected, a significant reduction in paw withdrawal thresholds has been observed (PWT; Figure 1) reaching a peak by D7 that continued up to D15 (the end of our experiment) post PCT (Figure 1).

Recently, we demonstrated that BPF administration inhibits the onset of neuropathic pain [39] and morphine-induced tolerance and hyperalgesia model [28]. In this study, we observed that intraperitoneal administration of BPF (25 mg/Kg), 15 min before paclitaxel administration, modified nociceptive parameters as follows: A significant enhancement in the mechanical and thermal threshold were produced in the presence of BPF starting from D7 up to D15 post PCT administration. (Figure 1A,B).

### 2.2. BPF Effects on Spinal Malondialdehyde (MDA) Levels during Paclitaxel-Induced Neuropathic Pain

**Figure 2:**
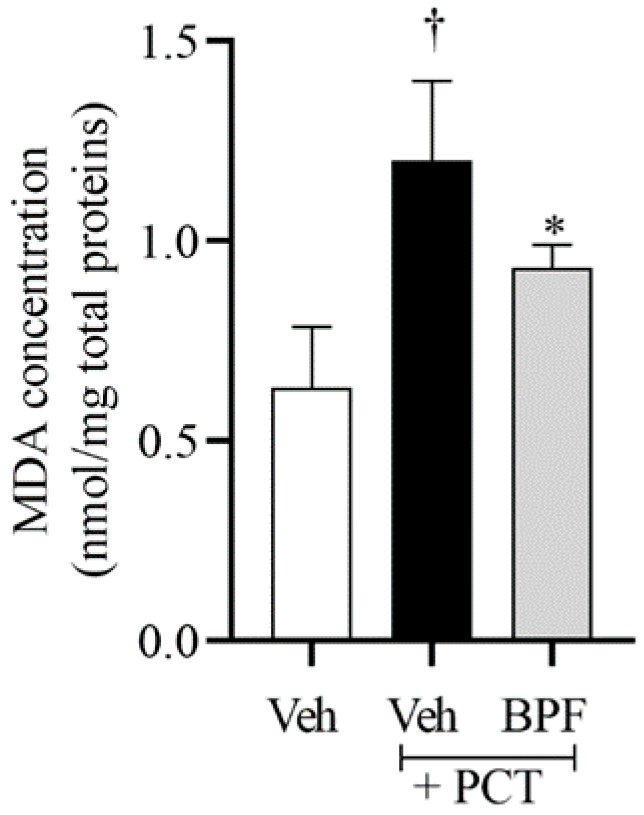
Beneficial effect of BPF on MDA levels. Compared to the vehicle group (Veh), animals with paclitaxel, at D15, showed a significant level of MDA in the L4-L5 portion of the spinal cord tissues. Intraperitoneal (i.p.) administration of BPF (25 mg/Kg/day; 15 min prior to each paclitaxel injection), led to a significant reduction of MDA levels. Results are expressed as mean ± SEM for 6 rats; †: *p* < 0.05 compared to Veh + Veh. *: *p* < 0.05 compared to Veh + PCT.

The development of neuropathic pain is associated with both activation of NOS enzymes and NADPH oxidase, and the production of superoxide (SO) in the spinal cord of rats [25]. The activation of NOS and NADPH oxidase is crucial for the biosynthesis of peroxynitrite (PN), responsible for protein nitration and formation of lipid peroxidation products, including MDA [25]. Indeed, in accordance with our previous data [25,39], we showed a significant MDA production in the spinal cord of rats that received PCT, suggesting an increase in lipid peroxidation products and oxidative stress (Figure 2). Intraperitoneal injection of BPF (25 mg/Kg), 15 min before paclitaxel injection, decreased MDA levels, demonstrating its antioxidant activity (Figure 2).

### 2.3. BPF and Post-Translational Modification of MnSOD during Paclitaxel-Induced Neuropathic Pain

Paclitaxel-induced neuropathic pain was associated with nitration of proteins in the dorsal horns of the spinal cord (L4-L5), including MnSOD.

**Figure 3:**
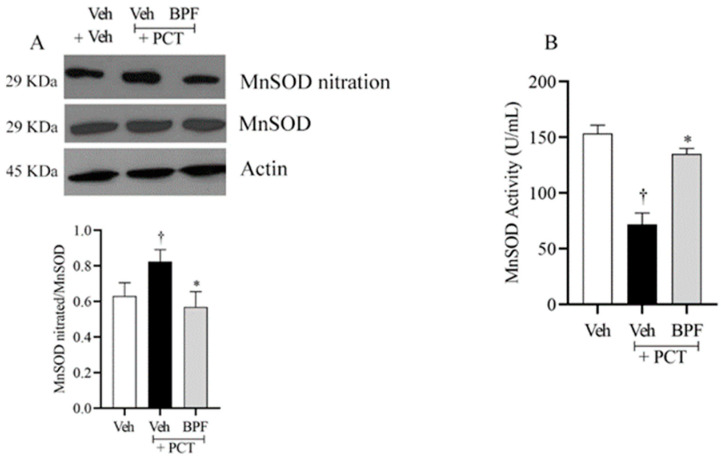
Beneficial effect of BPF on MnSOD nitration. (**A**) Compared to the vehicle group (Veh), animals with paclitaxel, at D15, showed nitration of manganese superoxide dismutase (MnSOD), (**B**) linked to the deactivation of its enzymatic function, in the L4-L5 portion of the spinal cord tissues. (**A**,**B**) BPF administration (25 mg/Kg/day; 15 min prior to each paclitaxel injection) inhibited MnSOD nitration and restored its activity in rats with paclitaxel-induced neuropathic pain. No difference in MnSOD or β-actin expression was detected among the lanes in these conditions. The gels are representative of results from six animals, and the histogram represents densitometric analyses of all animals per group. Results are expressed as mean ± SEM for six rats; †: *p* < 0.05 compared to Veh + Veh. *: *p* < 0.05 compared to Veh + PCT.

MnSOD nitration in the spinal cord contributes to the development of central sensitization in several experimental pain models [18,55], and this phenomenon also occurs in paclitaxel-induced neuropathic pain as shown in Figure 3A. Furthermore, MnSOD nitration in the rat’s spinal cord was associated with a significant loss of its ability to enzymatically dismutase the SO anion (Figure 3B). Intraperitoneal injection of BPF (25 mg/Kg), 15 min before paclitaxel injection, inhibited MnSOD protein nitration and restored MnSOD activity, thus re-establishing cellular oxidative homeostasis (Figure 3).

### 2.4. BPF and Post-Translational Modification of Gs and Glt-1 during Paclitaxel-Induced Neuropathic Pain

**Figure 4:**
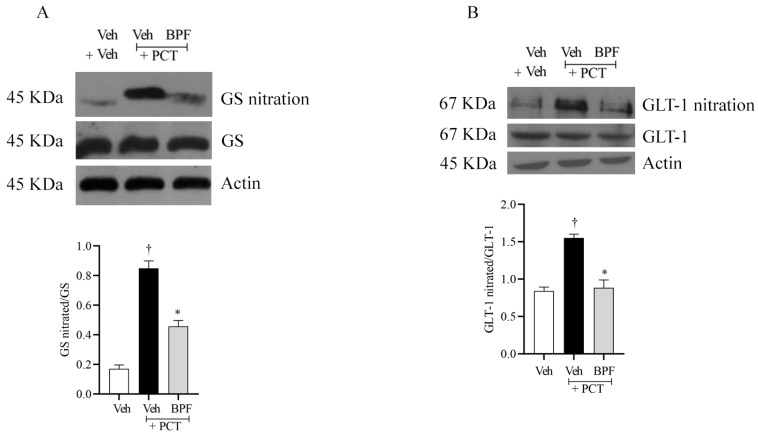
Beneficial effect of BPF on GS and GLT1 nitration. (**A**) Compared to the vehicle group (Veh), animals with paclitaxel, at D15, showed nitration of glutamine synthase (GS), and (**B**) glutamate transporter (GLT-1), in the L4-L5 portion of the spinal cord tissues. (**A**,**B**) Pretreatment with BPF (25 mg/Kg/day; 15 min prior to each paclitaxel injection) inhibited GS and GLT-1 nitration in rats with paclitaxel-induced neuropathic pain. No difference in GS, GLT-1 or β-actin expression was detected among the lanes in these conditions. The gels are representative of results from six animals, and the histogram represents densitometric analyses of all animals per group. Results are expressed as mean ± SEM for six rats; †: *p* < 0.001 compared to Veh + Veh. *: *p* < 0.001 compared to Veh + PCT.

We observed that paclitaxel-induced neuropathic pain was associated with nitration of glutamine synthase (GS), and glutamate transporter (GLT-1) (Figure 4).

Nitration of the astrocytic proteins GLT-1 (Figure 4B) and GS (Figure 4A), both responsible for regulating glutamatergic signaling, occurred in the spinal cord of paclitaxel-treated rats, most probably, contributing to the development and maintenance of neuropathic pain.

Intraperitoneal injection of BPF (25 mg/Kg), 15 min before paclitaxel injection, inhibited GS and GLT-1 protein nitration, thus re-establishing cellular oxidative homeostasis (Figure 4).

## 3. Discussion

Cancer treatment with chemotherapy drugs represents one of the factors that could influence the development of CIPN. In addition, analgesics used for neuropathic pain syndrome often show side effects and do not provide relief from CIPN [1]. This study demonstrated the beneficial effects of BPF administration in chemotherapy-induced neuropathic pain in rats. Neuropathy caused by chronic chemotherapy, commonly accompanied by neuropathic pain, is a severe dose-limiting complication associated with several first-line chemotherapy agents [1].

One of the main chemotherapeutic agents used is taxanes, including paclitaxel. Paclitaxel-induced neuropathic pain is known to be a dose-limiting side effect; although its mechanism is not yet fully understood, it is associated with hypersensitivity to mechanical and thermal stimuli that develop early in treatment initiation and persist for weeks or years after the end of treatment [37].

The resulting hyperalgesia and allodynia are associated with a persistent state of peripheral sensitization which consequently initiates the spinal sensitization. Here, we confirmed that paclitaxel injection, for 15 alternate days, produced thermal hyperalgesia and mechanical allodynia with a peak from day 7 to day 15 after paclitaxel administration. In addition, we showed that hyperalgesia and allodynia were associated with the increase of MDA levels (biochemical marker of lipid peroxidation and oxidative stress), attenuated by treatment with BPF at 25 mg/Kg.

The mechanisms involved in the development and maintenance of chronic neuropathic pain are still poorly understood, but several data support that ROS/RNS accumulation occurred after a central or peripheral nervous system injury could lead to the development of oxidative and nitrosative stress, neuronal cytotoxicity, and mitochondrial dysfunction, playing a crucial role in the pathogenesis of neuronal damage and degeneration [12,39].

Moreover, our previous data demonstrated the critical role of oxidative and nitrosative species, including peroxynitrite, for the development of peripheral and central sensitization associated with inflammatory and neuropathic pain [20,22,25].

Specifically, it has been observed that this condition led to an increase in post-translational modifications of key proteins involved in the maintenance of cellular oxidative homeostasis.

Here, we observed that during the paclitaxel treatment there was increased peroxynitrite, which led to nitration and deactivation of MnSOD. Furthermore, the glutamate transporter GLT-1 and glutamine synthase nitration occurred in the rats’ spinal cord (L4-L5), important events for the modulation of glutamate turnover and the central sensitization regulation.

Evidence showed that neuroinflammatory processes are due to impaired glia-neuronal communication. During CIPN, long-term activation of glial cells (astrocytes, microglia, Schwann cells), involvement of cytokine signaling, and changes in glutaminergic transmission have been well documented [36,58,59]. Specifically, chemotherapeutic drugs administration increases the release and production of the glial-derived proinflammatory cytokines responsible for neural cytotoxicity [36]. This mechanism, accompanied by an increase in oxidative stress is also responsible for the alteration of the glial transpostrator and glutamate functionality [25,59]. Obviously, it should be remembered that these mechanisms have been found not only in CIPN but also in inflammatory, neuropathic pain and morphine-induced tolerance, and hyperalgesia [15,28,38,55,59,60].

Glutamate is a crucial neurotransmitter involved in the pain development of several etiology, including neuropathic pain [61]. The extracellular glutamate concentration is essential for receptors activation and neurons protection from excitotoxicity and, it must be regulated to guarantee correct neuronal activity. Glutamate transporters (GLTs) in the glia and neurons plasma membranes have the role of removing glutamate from the extra-synaptic region and synapse itself. 90% of total glutamate transport is due to GLTs in glia [62].

When they are nitrated and then inactivated, the synaptic transmission results modified due to an increased glutamate concentration. Considering their involvement in cysteine reuptake, GLTs are also important in the biosynthesis of glutathione (GSH) which defends cells from oxidative stress and neuronal degeneration. On top of that, PN can inactivate, via nitration, the glutamate synthetase (GS), which catalyzes the conversion of ammonia and glutamate to glutamine [63]. Inactivation of GS seems to be involved in a different kind of pain. Thus, PN can alter glutamatergic signaling leading to central sensitization [15,28,63,64,65]. The prevention of tyrosine nitrations inhibits neuropathic and inflammatory pain, tolerance, and morphine-induced hyperalgesia thanks to ROS scavenging [28,65,66].

Recently, the use of natural products or plant derivatives has been more and more taken into consideration for the treatment of chemotherapy-induced neuropathy due to their antioxidant effect [67].

Studies showed that pharmacological inhibition of the synthesis of NO, SO, and PN can treat the pain of different etiology [25,28,34,55,66]. Therefore, the identification of free radical scavengers supports the idea that SO/PN is a valid therapeutic target for pain treatment.

In particular, the effect of the polyphenolic fraction of bergamot (BPF) on tolerance to morphine in mice and neuropathic pain in rats has been evaluated, highlighting both the antioxidant and anti-inflammatory properties of bergamot [28,39].

We also demonstrated BPF’s protective effect in CIPN. BPF administration, 15 min before the intraperitoneal injection of paclitaxel, ameliorated the neuropathic pain conditions assessed by measuring the mechanical allodynia and thermal hyperalgesia. In addition, we observed that BPF administration was able to inhibit the nitration of MnSOD, GS, and GLT-1. Prevention of ROS noxious effects by natural antioxidants employment prevents the development of neuropathy by inhibiting the hyperalgesic condition mediated by post-transductional modifications of tyrosine residues of endogenous antioxidant system and glutamatergic system at the level of the spinal cord.

## 4. Materials and Methods

### 4.1. Animals

Sprague-Dawley males’ rats (eight weeks old, Envigo), were used according to the European Economic Community regulations 2010/63/UE (authorization number 577-2016-PR), the Italian regulations for animal protection in experimental and other scientific purposes (D.L. 26/2014), and the NIH Guideline on Laboratory Animal Welfare, approved on 8 June 2016. A number of animals were used, necessary to obtain statistical significance at *p* < 0.05 as established by the guidelines of the International Society for the Study of Pain guidelines. Rats (*N* = 2) were housed per cage and kept in stable conditions of temperature (21 ± 1 °C), humidity (60 ± 5%) and controlled environment, with 12-h light/dark cycle and food and water ad libitum. Experiments were performed between 7:00 and 10:00 in a quiet room.

### 4.2. Natural Drugs

Natural antioxidant Bergamot polyphenolic fraction (BPF) was provided by H&AD (Herbal and Antioxidants Derivatives srl, Calabria, Italy. The specification sheet with the most relevant active ingredients of BPF is displayed in the Supplementary Material (Figure S1). BPF was obtained as previously described [51]. HPLC was performed to analyze BPF powder for polyphenol contents. Moreover, toxicological analyses excluded the presence of any toxic compound. No bacteria or mycotoxin were detected in microbiological standard tests.

Unless specified, all drugs were purchased from Sigma Aldrich (Milan, Italy) and dissolved in saline (sodium chloride 0.9%).

### 4.3. Experimental Group

Animals were randomly divided into the following groups:

Vehicle group: Rats (*n* = 15) received an intraperitoneal (i.p.) injection of saline 15 min before intraperitoneal (i.p.) injection of saline (vehicle), on alternate days, until D15 (D0, 3, 5, 7, 10, 12, 15).

Paclitaxel group: Rats (*n* = 15) received an intraperitoneal (i.p.) injection of saline 15 min before intraperitoneal (i.p.) injection of paclitaxel (PCT) (2 mg/Kg/die), on alternate days, until D15 (D0, 3, 5, 7, 10, 12, 15).

Bergamot groups: Rats (*n* = 15) treated with polyphenolic fraction of bergamot (BPF) (5, 25 or 50 mg/Kg/die) 15 min before intraperitoneal (i.p.) injection of paclitaxel (PCT) (2 mg/Kg/die), on alternate days, until D15 (D0, 3, 5, 7, 10, 12, 15).

The dose and the timing of BPF or PCT administration were chosen according to the literature [25,28,39]. For all groups, on day 15 (D15) rats were sacrificed and spinal cord (L4–L6) were explanted. The tissue was immediately frozen in liquid nitrogen and stored at −80 °C for subsequent analyses.

### 4.4. Behavioral Test

Test compounds were administered from day 0 (D0) (15 min before the first intraperitoneal injection of the chemotherapeutic agent) to D15. Each rat was allowed in a Plexiglas cage to acclimate for ∼20 min. If the test coincided with the day of drugs administration, the behavioral tests were performed before the drug was injected.

Mechanical allodynia was measured by evaluating rat paw withdrawal thresholds (PWT), through the use of Von Frey filaments (Ugo Basile, Varese, Italy), sequentially increasing and decreasing the stimulus strength (the “up and down” method) of a calibrated set of von Frey filaments. Each filament was applied (three times for the duration of two second) perpendicularly to the plantar surface in an increasing order of bending force sufficient to bend the microfilament. A positive response was defined as PWT, when rats showed at least three withdrawal responses out of five times application to a filament [68].

Hargreaves’s protocol [69] was used in order to evaluate damage in non-responsive animals’ tissues. The thermal stimulus was direct to a single hind paw (cut off latency of 20 s) through high intensity projector bulb (mobile unit). The test ended when the animal failed to respond by 20 s. Paclitaxel treatment results in bilateral hyperalgesia [70]. Data obtained from both left and right hind paws did not differ in any group or at any timepoints. Paw-withdrawal latency (sec) was used to present the results.

### 4.5. Tissue Preparation for Protein Extraction

Spinal cord (L4-L6) was homogenized with lysis buffer (20 mM Tris-Base; 150 mM NaCl; 10% Glycerol; 2 mM EDTA; 1% CHAPS; 1% protease inhibitor cocktail (*v/v*; Sigma, cat. 8340) at 1:3 *w/v* ratio. Therefore, 14,000 rpm centrifuge (4 °C, 45 min) was performed to obtain supernatants from solubilized extracts, and then stored at −80 °C until usage. Bicinchoninic Acid (BCA) protein assay (Thermo Scientific, cat. 23225, Milan, Italy) was used to determine protein concentration.

### 4.6. Western Blotting e Immunoprecipitation

Immunoprecipitation and western blot analyses were performed with the previous obtained proteins. For immunoprecipitation, 50 µL of protein A-sepharose 4B resin (Sigma, cat. P3391) was prepared through four PBS washes. Each time, resin was mixed and centrifuged with fresh PBS (14,000 rpm at 4 °C for 1 min). Lysis Buffer (20 mM Tris-Base; 150 mM NaCl; 10% Glycerol; 2 mM EDTA; 1%CHAPS; 1% protease inhibitor cocktail (*v/v*; Sigma, cat. 8340) were used to re-suspend the resin. Specific antibodies against the proteins of interest (anti-MnSOD, Millipore, cat. 06984; anti-GS, BD Biosciences, cat. 610517; or anti-GLT1, USBiological, cat. G4005-91, Swampscott, MA, USA) were coated and mixed overnight (O/N) at 4 °C to the resin. 300 μg of sample supernatant were added and incubated (O/N; 4 °C) with pre-washed resin. After centrifugation (14,000 rpm, 20 min, at 4 °C) the obtained pellets were collected for SDS-PAGE analysis. Immunoprecipitated protein complex and total lysates were used for western blot analyses and incubated with specific antibodies. 12% or 7.5% SDS-PAGE minigels were used to resolve immunoprecipitated proteins, and then transferred to nitrocellulose membranes. Membranes were blocked at room temperature in 1% BSA/0.1% thimerosal in 50 mM Tris−HCl (pH 7.4)/150 mM NaCl/0.01% Tween-20 (TBS/T), for 1 h. Then, membranes were incubed at 4 °C, O/N with: anti-GS (1:1000); anti-MnSOD (1:1000); anti-GLT-1 (1:1000), washed with TBST and incubated for 1 h at room temperature with anti-mouse (1:10,000; GE Healthcare, cat. NA931) or anti-rabbit (1:15,000; GE Healthcare, cat. NA934) secondary antibodies conjugated to horseradish peroxidase. Enhanced chemiluminescence (ECL; GE Healthcare, cat. RPN2232) was used to visualize the protein, after washing. After stripping the membrane, with buffer solution (Thermo scientific, cat. 21059), actin and GS levels were detected following standard protocol. Among the lane, no difference for the actin (1:5000, Sigma, cat. A3853) was detected. Thus, for each lane densitometry data were normalized against actin and expressed as the ratio of nitrated to non-nitrated proteins. ImageQuant 5.2 software (Molecular Dynamics) was used to determine the quantitation of protein bands by densitometry.

### 4.7. MnSOD Activity

Superoxide Dismutase Assay kit (Cayman Chemical) was used to visualize MnSOD activity, following standard protocols. Evaluation of superoxide radicals, generated by xanthine oxidase and hypoxanthine, was performed with tetrazolium salt. Standard curve was generated with a quality-controlled SOD standard.

TECAN Sunrise Reader (Tecan, Männedorf, Switzerland) was used to measure absorbance at 440–460 nm. Enzymatic activity was presented as units/mL. One unit of SOD is the amount of enzyme needed to exhibit 50% dismutation of the superoxide radical. The results were defined after triplicate analysis.

### 4.8. Malonylaldehyde Assay

MDA detection was performed by measuring thiobarbituric acid reactive substances (TBARS). Lysate, after homogenization with 10% NaOH, 20% Acetic Acid and TBA, were boiled at 95 °C for 1 h and placed on ice for 10 min. Then, samples were centrifuged for 10 min at 1600× *g* at 4 °C, loaded into a black 96-well microtiter plate, and fluorometrically measured at an excitation wavelength of 530 nm and emission wavelength of 550 nm, using Infinite 200 microplate fluorometer (Tecan, Männedorf, Switzerland,).

### 4.9. Statistical Analysis

The Kolmogorov–Smirnov test was used for analysis of the data distribution. After confirmation of normal data, differences between groups were compared by analysis of variance (ANOVA). Two-way repeated measures ANOVA with Bonferroni comparisons were used for data obtained from each time point. Other data were analyzed via one-way ANOVA followed by the Newman–Keuls test. Statistical significance was fixed at *p* < 0.05. The results are expressed as mean ± SEM. Analyses were carried out using GraphPad Prism software (v8.00; GraphPad Software, Inc., San Diego, CA, USA).

## 5. Conclusions

In conclusion, our results provide information necessary to confirm that bergamot could be considered an important therapeutic agent capable of limiting the toxicity and therefore the development of neuropathic pain induced by paclitaxel, without hindering its beneficial anticancer effects.

## Data Availability

The data presented in this study are available on request from the corresponding authors.

## Supplementary Materials

The following are available online at https://www.mdpi.com/article/10.3390/ph14100975/s1, Figure S1: BPF: Bergamot Polyphenolic Fraction Specification sheet.
