# Supplementary material for: The Protective Effect of Bergamot Polyphenolic Fraction (BPF) on Chemotherapy-Induced Neuropathic Pain
Source: Pharmaceuticals (Basel). 2021 Sep 25;14(10):975. doi: 10.3390/ph14100975 (PMC8540578; doi:10.3390/ph14100975)
Supplement: Supplementary file 1 [file pharmaceuticals-14-00975-s001.zip › pharmaceuticals-1349441-supplementary.pdf]

Supplemental Material
Figure S1
BPF: Bergamot Polyphenolic Fraction
Specification sheet

DESCRIPTION	SPECIFICATIONS	METHODS
Botanical Source	**Citrus Bergamia Risso et Poit.**	
Family	**Rutaceae**	
Synonyms	**Citrus aurantium var. bergamia**	
Country of Origin	**Calabria, Italy**	
Part Used	**Fruit**	
Shelf Life	**3 years, if correctly stored**	
**ORGANOLEPTIC**		
Colour	**Yellow Powder**	**visual (CQ-MO-148)**
Odour	**Aromatic**	**visual (CQ-MO-148)**
Flavour	**Characteristic of bergamot**	**sensory (CQ-MO-148)**
**CHEMICAL CHARACTERISTICS**		
pH	**3.0 – 4.0**	**IM (0.5% in water) at 25°C**
Average Mesh Size	**Pass 70 mesh**	**Sieve: (CQ-MO-023)**
Bulk Density	**30-70g/100ml**	**PT CHIM 65 rev 0 2011**
Moisture Content	**< 10.0%**	**ISTISAN 96/34, pag 7**
Organic Solvent Residue	**None**	**GC: (CQ-MO-168)**
Soluble in 40°C H_2O	**Good**	**visual: (CQ-MO-148)**
Soluble in 50% H_2O + EtOH	**Good**	**visual: (CQ-MO-148)**
Active Ingredient Strength	**HPLC**	
Pesticides Residue	**Negative**	**PT CHIM 69rev 02 011**
**ACTIVE INGREDIENTS**	**UNIT**	**RANGE**
Polyphenols (Neoeriocitrin, Naringin, Neohesperidin, Melitidin, Bruteridin)	%	**38%**

**HEAVY METALS**		
Arsenic	ppm	**<2.0**
Lead	ppm	**<2.0**
Heavy Metals (tot. amount)	ppm	**<20.0**

**MICROBIOLOGICAL EVALUATION**		
Aerobic Plate Count	**<1,000 CFU/g**	**ISO 4833-1:2013**
Yeast and Mold Count	**<100 CFU/g**	**ISO 21527-1:2008**
E. Coli	**Negative**	**ISO 16694-2:2001**
Coliform	**Negative**	**ISO 4832:2006**
Salmonella	**Negative**	**UNI EN ISO 6579:2000**
Staphylococcus Aureus	**Negative**	**UNI EN ISO 6888-2:2004**
Streptococci	**Negative**	**PT BAT26 rev0 02012**
**PRODUCT TREATMENT**		
Extraction solvents	**Water+KOH**	
Drying Method	**Sprydry**	
